# The burden of COVID-19 infection in a rural Tamil Nadu community

**DOI:** 10.1186/s12879-021-06787-0

**Published:** 2021-10-28

**Authors:** R. Isaac, B. Paul, M. Finkel, M. Moorthy, S. Venkateswaran, T. T. Bachmann, H. Pinnock, J. Norrie, S. Ramalingam, S. Minz, S. Hansdak, R. Blythe, M. Keller, J. Muliyil, D. Weller

**Affiliations:** 1 Christian Medical College, Vellore, Tamil Nadu India; 2 Centre for Population Health Sciences, Usher Institute, University of Edinburgh, Edinburgh, EH8 9AG UK; 3 Weill Cornell Medical College, New York, USA; 4 Infection Medicine, Biomedical Sciences, University of Edinburgh, Edinburgh, UK

**Keywords:** COVID-19, Rural, Tamil Nadu, India

## Abstract

**Background:**

There have been over 30 million cases of COVID-19 in India and over 430,000 deaths. Transmission rates vary from region to region, and are influenced by many factors including population susceptibility, travel and uptake of preventive measures. To date there have been relatively few studies examining the impact of the pandemic in lower income, rural regions of India. We report on a study examining COVID-19 burden in a rural community in Tamil Nadu.

**Methods:**

The study was undertaken in a population of approximately 130,000 people, served by the Rural Unit of Health and Social Affairs (RUHSA), a community health center of CMC, Vellore. We established and evaluated a COVID-19 PCR-testing programme for symptomatic patients—testing was offered to 350 individuals, and household members of test-positive cases were offered antibody testing. We also undertook two COVID-19 seroprevalence surveys in the same community, amongst 701 randomly-selected individuals.

**Results:**

There were 182 positive tests in the symptomatic population (52.0%). Factors associated with test-positivity were older age, male gender, higher socioeconomic status (SES, as determined by occupation, education and housing), a history of diabetes, contact with a confirmed/suspected case and attending a gathering (such as a religious ceremony, festival or extended family gathering). Amongst test-positive cases, 3 (1.6%) died and 16 (8.8%) suffered a severe illness. Amongst 129 household contacts 40 (31.0%) tested positive. The two seroprevalence surveys showed positivity rates of 2.2% (July/Aug 2020) and 22.0% (Nov 2020). 40 tested positive (31.0%, 95% CI: 23.02 − 38.98). Our estimated infection-to-case ratio was 31.7.

**Conclusions:**

A simple approach using community health workers and a community-based testing clinic can readily identify significant numbers of COVID-19 infections in Indian rural population. There appear, however, to be low rates of death and severe illness, although vulnerable groups may be under-represented in our sample. It’s vital these lower income, rural populations aren’t overlooked in ongoing pandemic monitoring and vaccine roll-out in India.

## Background

The coronavirus disease (COVID-19) pandemic caused by the severe acute respiratory syndrome coronavirus-2 (SARS-CoV-2) began in early 2020; there are now over 200 million cases worldwide and over 4.3 million deaths [1]. In India, the world’s second most populous nation, there was a rapid rise in cases from the onset of the pandemic in March 2020, mostly from the urban areas, yet there was relatively little information on the infection rate in the rural areas [2, 3]. Concerns were expressed at an early stage that COVID-19 infection could be spreading unchecked in rural areas of India, where 70% of the population reside, because of disparities in access to and compliance with preventive measures and inadequate health information systems [4]. While spread of the virus in India was initially traceable to known sources of infection (such as international travellers), community transmission soon became established, with no clear identifiable source of the infection [5]. The second wave of the pandemic in India, around March to June 2021, had a deadly impact, with more virulent strains of the virus and more young people affected [6]. Available evidence shows that there is much variation in transmission rates from region to region in India, depending upon factors including the susceptibility of the population, population density, and feasibility and uptake of COVID-19 preventive measures [7, 8]. As of August 13th 2021, there have been over 32.1 m cases of COVID-19 reported in India with over 430,000 deaths [9].

In the early stages of the pandemic (May–June 2020), the Indian Council of Medical Research (ICMR) undertook a cross sectional seroprevalence survey in selected districts, stratified by incidence rate; it reported a pooled adjusted seroprevalence of 0.73% (0.34–1.13%) amongst adults residing in both rural and urban areas [10]. The second seroprevalence survey undertaken by ICMR between August 17 and September 22, 2020 examined the prevalence in rural and urban areas separately; it reported a pooled prevalence of 7.1% (95% CI: 6.2–8.2) with 4.4% in rural populations [11].

With increasing rates of COVID-19 infection in the country, the Government of India (GoI) initiated multiple preventive measures, in coordination with the state governments. These included lockdown, active airport screening, quarantining, recommendations for ‘work from home’, closure of educational institutions, limiting public transport, public awareness campaigns, and active case detection with contact tracing in most regions [12]. At the time of our study (commencing June 2020) there were lockdown conditions; after July 2020 these were eased, but some restrictions for large gatherings, recreation and religious gatherings remained. Schools in rural Tamil Nadu had little online provision, and remained closed longer than many city schools. Compliance with the use of masks was not high in these rural areas and, at village level, social distancing wasn’t well-observed. COVID-19 vaccines hadn’t been introduced in India during our study period.

The GoI also launched a nation-wide testing programme for symptomatic individuals using RT-PCR tests, which was done through district hospitals and primary health care centers. The measures were largely in line with the WHO’s Strategic Preparedness and Response Plan for COVID-19 [13]. While this initiative increased the access to detection of cases in the rural areas, there were problems with implementation, and there was limited information available about the burden of the disease among rural communities.

To address this information gap, we undertook a study that examined the burden of infection in a rural population of the Vellore district, Tamil Nadu. We:

1. established and evaluated a COVID-19 PCR-testing programme in a rural population in the Vellore, Tamil Nadu district;
2. initiated a COVID-19 seroprevalence survey in the same community.

We combined our results with secondary data to produce estimates of total numbers of infections in our population, and infection-to-case ratios (ICRs), a measure of how well cases might predict actual infections [14]. We also examined the effect of socio-demographic characteristics and other risk factors on COVID-19 test-positivity in both study components.

## Methods

### Study setting and study population

The study was undertaken by an international team led by the Rural Unit of Health and Social Affairs (RUHSA), a community health center of CMC, Vellore, with long-established programmes of preventive activities [15]. RUHSA provides health care services primarily to residents of the ‘KV Kuppam community development block’ (an administrative unit) and, to a limited extent, to those in the neighbouring blocks of Vellore district, Tamil Nadu (Gudiyatham, Madhanur and Peranampattu rural revenue blocks) [16]. The KV Kuppam is a rural block with a population of almost 130,000 people, and is divided into 18 peripheral service units (PSUs) under the RUHSA health programme, comprising 3–4 villages with a sub-centre in each PSU.

Residents of the rural villages in the study catchment area are primarily engaged in agriculture; other occupations include small businesses, weaving, poultry farming and salaried service jobs. Literacy levels among men are about 77% and among women 64%—consistent with findings from other similar regions in India [17]. Typical rural housing layouts are rows of terraced, single storied houses; apart from houses at the end of the terrace, the only open spaces are in the front and the back of each row forming the village streets. Residents typically socialise by sitting in front of their houses facing the streets, potentially exposing passers-by to COVID-19 virus infected individuals. Additionally, prior to our study, there was an influx of migrant workers who were employed in neighbouring cities, and who were returning home due to the pandemic crisis and lockdown (this, too, is a source of concern for disease transmission [18]). We undertook two studies in separate populations within our catchment area:

1. COVID-19 testing programme
  *Recruitment* We used the PSUs as the basis of our recruitment strategy. Trained community health workers (CHWs) from RUHSA, with the assistance of village leaders, provided the villagers with information on COVID-19, including common symptoms. Individuals who received this information and thought that they may have COVID-19-related symptoms were invited to attend a ‘fever clinic’ at RUHSA where they completed a screening questionnaire [19, 20] (see “Appendix 1”). Those who met the criteria were offered PCR-testing, and if they tested positive were classified as (1) *Minor*—home quarantined and didn’t receive oxygen or whose admission period was uneventful, (2) *Moderate*—patients who required admission with oxygen support but not ICU care or (3) *Severe*—those who required oxygen and ICU CARE.
  *Study details* The fever clinic was established at RUHSA beginning June 8 2020. After informed consent was obtained, attendees were interviewed to collect sociodemographic characteristics, 14-day travel and contact history, and history of relevant symptoms. A clinical examination was performed and vital signs including pulse rate, respiratory rate, oxygen saturation, temperature and body mass index (BMI) were recorded. Nasopharyngeal samples were collected by trained lab technicians using Dacron-flocked swabs, following recommended preventive measures [21]. Swabs were placed in a viral transport medium, safely packed, with adequate cold chain maintenance, and transported on the same day to the virology laboratory at CMC, Vellore. We undertook telephone follow-up of the participants—we obtained their mobile phone numbers at the time of presentation. They were called a minimum of 3 times, 3 months after their diagnosis. *Data analysis:* Sociodemographic characteristics of the study participants, contact history, comorbidities, travel history and history of participation in gatherings, common symptoms and severity of disease at first presentation to the clinic were recorded.

Box 1 Test characteristics and procedures: RT-PCR testing programme

- *In the laboratory, the samples were processed as batches in class IIB biosafety cabinets (BSC). Staff were wearing appropriate personal protective equipment*
- *Briefly, swabs were vortexed and an aliquot stored at *− *80 °C. Nucleic acid was extracted in a QIAcube HT, (Qiagen Inc) an automated nucleic acid extraction platform as per manufacturer’s instructions. Extracts were used for RT-PCR to detect SARS-CoV-2 RNA using the Altona Realstar assay (Altona Diagnostics GmbH).*
- *The Altona Realstar assay, a multiplex real-time RT-PCR assay, uses primers and probes specific to the envelope (E) and spike (S) genes of SARS-CoV-2 along with an internal control.*
- *Assays were validated as per manufacturer’s instructions and cycle threshold (Ct) values noted for E and S-gene targets.*
- *When a single gene positivity (E or S) was observed, a repeat sample was requested for result confirmation of the result.*

2. Seroprevalence study
  The details of our seroprevalence surveys are shown in Table 1. Seroprevalence surveys 1 and 2 sought to obtain estimates of community prevalence of COVID-19 antibodies, and used random sampling from the RUHSA database of enrolled patients. They were conducted 4 months apart; the first survey included children, while the second only recruited adults aged 18 or over. The family contact seroprevalence survey was conducted in households of individuals who tested positive at our fever clinic, to obtain an estimate of within-household transmission.

**Table 1:** Seroprevalence surveys—details

	Timing	Sampling strategy	Target group
Seroprevalence survey 1	July 6th to August 20th, 2020	150 randomly selected households (15 households from each of 10 randomly selected clusters)	All individuals above 5 years of age
Seroprevalence survey 2	November 16th to 20th 2020	20 randomly selected households (20 households from each of 10 randomly selected clusters)	All adults 18 years of age or over
Family contact seroprevalence survey	July 6th to September 15th 2020	Approached all households 14 days after the diagnosis in the fever clinic index case; sampled consenting family contacts but excluded those who were known to be RTPCR positive	Individuals living in households of PCR-positive participants from fever clinic, including children above 5 years—788 contacts identified

Box 2 Test characteristics and procedures: Seroprevalence study

- *The seroprevalence of COVID-19 infection was measured by testing for IgG antibodies.*
- *We assumed that most cases develop detectable IgG antibodies against COVID-19 from 15 days post-infection*
- *There appears to be a differential response to the proteins of COVID-19 and also an association with the disease severity has been reported *[22]*. Therefore any positivity is indicative of exposure to the virus*
- *Blood samples were collected in Vacutainer tubes with clot activator (BD Cat# 367837, Becton Dickinson, Inc.). Samples were transported to the laboratory at ambient temperature.*
- *In the laboratory, an aliquot of serum was stored at − 80 °C until batch testing*
- *COVID-19 antibody detection was performed on two commercially available platforms—Elecsys Anti-SARS-CoV-2 on the Cobas e411 analyser (Roche Diagnostics Pvt. Ltd) and the SARS-CoV-2 IgG assay (COV2G) on the ADVIA Centaur system (Siemens Healthcare Private Limited)*
- *The Elecsys assay detected antibodies to SARS-CoV-2 Nucleoprotein antigen (anti-N) using an electrochemiluminescence (ECLIA) assay principle. The COV2G assay detects IgG antibodies against the spike 1-receptor binding domain (S1-RBD) of SARS-CoV-2 uses a sandwich chemiluminescent immunoassay principle. Both assays report a reactivity index where a value* ≥ *1 is considered positive and* < *1 considered negative for antibodies to COVID-19*

*Sample size* We sought prevalence estimates and estimates of precision; based on WHO guidance, our expected ‘margin of error’ corresponds to the expected width of the 95% confidence interval associated with our point estimate of ‘p’—calculated using the binomial likelihood method [23]. For our PCR-testing programme in the fever clinic, we estimated a prevalence of 25%—our sample size (350) provided 20% precision*.* We did not have any reliable estimate of the prevalence of COVID seropositivity in our population. We used a sample of 500 participants from 150 households—using estimates of 1% and 5% to provide a 0.76% and 3.65% margin of error respectively. For the 2nd survey, a sample of 200 participants would provide an 8.6% margin of error for an estimated prevalence of 10%. A sample size of 200 was calculated for the family contact survey, based on a predicted prevalence of seropositivity of 30% with 20% precision.

*Data analysis* Basic sociodemographic characteristics of the participants were described. COVID-19 infection rates, with associated 95% confidence intervals (CIs) were calculated for both the random community and family contact surveys. The proportion of confirmed cases among the attendees of the fever clinic with 95% confidence interval was computed. Factors associated with confirmed cases were compared with those who were negative among the study participants.

### Total cases and infection-to-case ratio

We made estimates of the total number of cases by extrapolating from our results using secondary data. These data were publicly available through the Tamil Nadu Government covid data portal [24]; we were able to obtain specific data for the KV Kuppam block (our study site) through the government’s Vellore district level centre. The infection to case ratio (ICR) was defined as the number of individuals with COVID-19 infection (in our seroprevalence survey) divided by the number of RT-PCR positive cases reported from the block documented by the government health system, 2 weeks before the date of seroprevalence sample collection (this was based on our 2nd survey data).

## Results

### COVID-19 testing programme

In our study population 602 individuals completed the screening questionnaire at the fever clinic of whom 350 met the criteria for RT-PCR testing. Of these 182 (52%, 95% CI: 46.8–57.2) tested positive for COVID-19. Table 2 shows patient characteristics and potential risk factors of those undergoing testing. Significant risk factors for a positive test were older age group, male gender, higher socioeconomic status (SES, as determined by occupation, education and housing), a history of diabetes, contact with a confirmed/suspected case and attending a gathering (such as a religious ceremony, festival or extended family gathering).

**Table 2:** Sociodemographic characteristics and potential risk factors for COVID-19 infection amongst fever clinic attendees—by test result

	Positive n = 182	Negative n = 168	p value
Age in Years—median (IQR)	44 (32, 56)	31.5 (19.3, 58.8)	< 0.001*
Gender (n male, %)	129 (70.9)	84 (50)	< 0.001*
Education (n, %)			
Less than high school	61 (33.5)	88 (52.4)	< 0.001*
High School and above	121 (66.5)	80 (47.6)	
Occupation (n, %)			
Homemakers, unemployed	58 (32)	95 (56.5)	< 0.001
Unskilled labourer, semiskilled	34 (18.6)	35 (20.8)	
Cultivation/farmer	6 (3.3)	6 (3.6)	
Business	28 (15.4)	9 (5.4)	
Professional	19 (10.4)	5 (3)	
Service jobs	37 (20.3)	18 (10.7)	
Housing facility (n, %)			
Poor housing (Thatched roof)	38 (19.9)	51(30.4)	0.042
Good housing (Concrete house)	144 (79.1)	117 (69.6)	
Smoker (n, %)	6 (3.3)	8 (4.8)	0.485
Travel history (n, %)	131 (72)	134(79.8)	0.090
Contact with confirmed/suspected case (n, %)	41 (22.5)	24(14.3)	0.048
Attended gathering in last 14 days (n, %)			
Death ceremony	67 (36.8)	32 (19)	< 0.001
Small community gathering	12 (6.6)	2 (1.2)	
Wedding	10 (5.5)	5 (3)	
Market	5 (2.7)	7 (4.2)	
Other	36 (19.8)	18 (10.7)	
Co-morbidities			
Chronic lung disease	9 (4.9)	13 (7.7)	0.282
Chronic Kidney, Liver, Heart disease	9 (4.9)	3 (1.8)	
Diabetes	42 (23.1)	20 (11.9)	0.006*
Hypertension	29 (15.9)	19 (11.3)	0.209
Immunosuppressive drugs	0 (0)	1 (0.6)	

Symptoms amongst participants are presented in Table 3; those most strongly associated with a positive test were fever with fatigue (p = 0.004), cough (p ≤ 0.001), loss of smell (p = 0.001) and loss of taste (p ≤ 0.001). Of the 182 participants who tested positive, at presentation 3 (0.9%) had ‘severe disease’ 13 (3.7%) had ‘moderate disease’ and rest of the 331 (95.4%) had minor illness.

**Table 3:** Symptoms amongst fever clinic attendees

	Positive n = 182	Negative n = 168	p value
Fever	154 (84.6)	138 (82.1)	0.534
Chills	49 (26.9)	50 (29.8)	0.556
Fatigue	128 (70.3)	93 (55.4)	0.004
Cough	108 (59.3)	63 (37.5)	< 0.001
Runny Nose	42 (23.1)	29 (17.3)	0.177
Loss of smell	55 (30.2)	17 (10.1)	< 0.001
Loss of taste	61 (33.5)	24 (14.3)	< 0.001
Shortness of breath	50 (27.5)	30 (17.9)	0.032
Sore throat	62 (34.1)	52 (31)	0.535
Diarrhoea	26 (14.3)	20 (11.9)	0.510
Nausea/Vomiting	33 (18.1)	42 (25)	0.118
Haemoptysis	2 (1.1)	1 (0.6)	0.610
Body Ache	107 (58.8)	78 (46.4)	0.021
Abdominal pain	23 (12.6)	21 (12.5)	0.969
Chest pain	11 (6)	7 (4.2)	0.427

The research officer undertook telephone follow-up, calling 179 out of 182 patients—3 had died. Of these, 51 didn’t respond to the calls, 41 had treatment at CMC, 16 had severe illness and 13 had moderate illness.

### Seroprevalence surveys

The sociodemographic characteristics of the participants in both serosurveys were similar, but not identical (Table 4); there was a slightly higher proportion of men and younger people in the first survey, but overall characteristics were consistent with existing descriptive data in our population [25]. About half of the participants were male, two-thirds had education up to high school and above, and one-third lived in a lower-income style of housing facility. As seen in Table 4, more than 80% of the participants gave a history of travel to neighbouring villages and towns related to work and only a small proportion gave a history of participating in social/community gatherings.

**Table 4:** Socio-demographic characteristics and other risk factors of the participants in seroprevalence surveys by test result

	Serosurvey1 (July 6th to August 20th)			Serosurvey 2 (November 6th to 20th)		
	Negative n = 490	Positive n = 11	*p* value	negative n = 156	positive n = 44	*p* value
Age in Years—median (IQR)	38 (19, 55)	48 (26, 54)	0.08	49 (40, 61)	46 (36.25, 60)	0.324
Gender (n males, %)	241 (49.2)	5 (45.5)	0.807	59 (37.8)	14 (31.8)	0.465
Education (n, %)						
Less than high school	73 (14.9)	1 (9.1)	0.591	28 (17.9)	13 (30.2)	0.078
High School and above	417 (85.1)	10 (90.9)		128 (82.1)	30 (69.8)	
Occupation (n, %)						
Homemakers, unemployed	277 (56.5)	4 (36.4)	0.144	88 (56.4)	23 (52.3)	0.638
Unskilled, semi-skilled labourer	117 (23.9)	3 (27.3)		43 (27.6)	13 (29.5)	
Business	23 (4.7)	0		6 (3.8)	2 (4.5)	
Professional	21 (4.3)	2 (18.2)		5 (3.2)	4 (9.1)	
Cultivation/Farmer	35 (7.1)	1 (9.1)		7 (4.5)	1 (2.3)	
Service jobs	17 (3.5)	1 (9.1)		7 (4.5)	1 (2.3)	
Housing (n, %)						
Poor housing	162 (33.1)	5 (45.5)	0.389	58 (37.2)	15 (34.1)	0.707
Good housing	328 (66.9)	6 (54.5)		98 (62.8)	29 (65.9)	
Smoker (n, %)	32 (6.5)	1 (9.1)	0.735	7 (4.5)	1 (2.3)	0.508
Travel history	420 (85.7)	9 (81.8)	0.716	130 (83.3)	38 (86.4)	0.628
Contact with confirmed case	6 (1.2)	0	0.712	7 (4.5)	0	0.153
Participated in gathering last 14 days	14 (2.9)	0		6 (3.8)	5 (11.4)	0.053
Co-morbidities						
Chronic lung disease/TB	10 (2.1)	0		6 (3.8)	3 (6.8)	0.401
Chronic Renal, liver, heart disease	4 (0.8)	0		2 (1.3)	1 (2.3)	
Diabetes	25 (5.1)	2 (18.2)	0.057	23 (14.7)	6 (13.6)	0.854
Hypertension	23 (4.7)	2 (18.2)	0.042	26 (16.7)	5 (11.4)	0.391

Of the 501 random community participants tested in serosurvey 1, 11 (2.2%, 95% CI: 0.8, 3.4) tested positive (of these 4 were positive for both tests, 6 were only Roche positive and 1 was only Siemens positive). Of the 200 participants in the second serosurvey, 44 (22%, 95% CI: 16.3–27.7) tested positive (of these, 30 were positive by both Roche and Siemens test, 8 by Roche alone and 6 by Siemens alone).

In our household contact seroprevalence survey, 129 (16.6%) of the 788 known exposed family contacts consented to provide blood samples. Of these 40 tested positive (31.0%, 95% CI: 23.02 − 38.98); 30 were positive for both Roche and Siemens tests, 2 were positive for Roche test alone and 8 were for Siemens test alone.

### The burden of COVID-19 infection in our study population

**Fig. 1:**
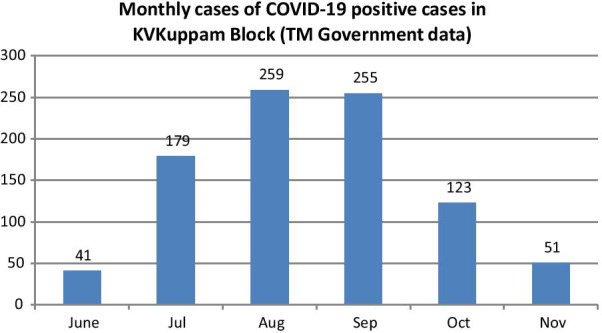
Monthly cases of COVID-19 positive cases in KV Kuppam Block (TM Government data)

Applying the prevalence of antibodies among the random community sample surveyed in the month of November 2020 (22%) to the total population of 128,679, we estimated a cumulative 28,300 infections in KV Kuppam rural block by November 2020. Based on a total of 858 cases reported in KV Kuppam block by 31st October 2020 (Fig. 1) [26], we estimated the infection to case ratio (ICR) to be 31.7 (95% CI: 31.1–32.3) up to that date.

## Discussion

To date there have been few studies reporting on the burden of the COVID-19 pandemic in rural Indian communities. Our study found that in a sample of our target population who met at-risk criteria for COVID-19, over half tested positive for the disease, confirming that the pandemic has become established in rural populations, despite their relative isolation. Further, our study is one of the first to examine COVID-19 seroprevalence in a rural area of India—this is crucial to understanding the likely extent of herd immunity developing and strategies for the containment of the pandemic [27].

Factors associated with test-positivity in our fever clinic survey were male gender, older age, higher SES, social interaction and presence of diabetes. This reflects the rather complex epidemiological profile of risk factors for COVID-19 and has been seen in other studies in the Indian population [28]. While lower income/deprived life circumstances may be more conducive to disease transmission, higher SES individuals may be more mobile, and more likely to come in contact with people from areas of India with high levels of COVID-19 (such as large cities). Nevertheless, in terms of impact, it is lower SES communities which are the most vulnerable, with most studies suggesting that COVID-19 is likely to worsen poverty and health disparities [29]. In our seroprevalence studies, we were not able to replicate the observed associations with test-positivity, but were limited by small numbers.

Only 3 (1.6%) out of the 182 fever clinic participants had died when we undertook telephone follow-up, and 16 (8.8%) had suffered a severe illness. This case fatality rate (CFR) is low in comparison with other international studies [30]; national data in India show CFRs among men of 2.9% and 3.3% among women [31]. It may indicate that, while the epidemic has taken hold in our rural population, its impact in terms of prolonged, severe illness and death, may be lower than in other, seemingly less vulnerable populations. However, while we are confident we captured all events in our fever clinic follow-up processes, our numbers are small. Also, CFRs are prone to bias; our fever clinic patients were symptomatic, and capable of travelling to the testing facilities at RUHSA. They were also a young population—even compared to population level data on Indian average age of COVID-19 infection, which show lower ages than in most western countries [41]. Hence, national, and international comparisons of CFRs should be treated with caution. Nevertheless, at a national level, India’s low case-fatality rates (particularly before the second wave) have attracted significant praise for the country’s public health efforts [32]. Numerous contributing factors have been proposed, including India’s younger population, cross-reactive immunity from other coronavirus infections in the past and environmental factors such as sunlight and vitamin D [33]. Cautious interpretation of available data on deaths and case fatality rates has, nevertheless, been urged—along with the avoidance of any degree of complacency [34]. Importantly, the Indian Government, despite the severe challenges of the pandemic’s second wave, has maintained its commitment to widespread testing and vaccine rollout.

Our seroprevalence findings showed a cumulative incidence of 2.2 percent by the end of August, increasing to 22 percent by November 2020, with an infection- to case-ratio of 33. This finding contrasts with the ICMR surveys which, by the end of September, showed lower seroprevalence (4.4% in rural populations) but, in the early stage of the pandemic, showed an ICR of 81.6 (95% CI: 48.3–141.4) [10, 11]. A seroprevalence study in Mumbai showed a rate of 54.1 percent in slums and 16.1 percent in non-slum areas of the city [35]. The estimates of seroprevalence were lower in this rural population compared to cities and urban slums in India. The factors underlying these differences are likely to be multifactorial, and include lower population density in our population and natural social distancing compared to cities and urban slums [36]. Our estimate of 33 infections for every confirmed case is commensurate with other studies which have examined ICR [14]—confirming that much infection remains clinically undetected in our study community, as in other parts of India.

Our estimates of seroprevalence of COVID 19 infection rates in rural India add to a growing body of international data. A pooled, worldwide estimate of 3.38% (95% CI 3.05–3.72%) was published in August 2020, but there was significant variation from country to country – for example, 5.27% (3.97–6.57%) in Northern Europe; 2.02% (1.56–2.49%) in Eastern Asia; and 1.45% (0.95–1.94%) in South America [37]. Regional and international comparisons do, however, need to be interpreted with caution—they are dependent on many factors, including timing of the surveys, population sampling and coverage. About one-third of household contacts of known cases were sero-positive—high in comparison to many other international studies [38]; in the presence of community control measures, transmission within households is thought to account for about 70% of COVID-19 infections [39].

There are a number of limitations to our study; crucially, it was undertaken before the pandemic’s severe second wave in India (March–June 2021)—and this has changed some of the patterns of disease transmission [40]. Our ‘fever clinic’ survey was prone to sampling bias; villagers self-selected in response to community health workers making them aware of the study and the possibility of being tested. Travel to the clinic at RUHSA meant time away from work or the household; although travel costs were reimbursed, it is possible that those with more severe symptoms, or who were more financially challenged, were less likely to attend. Nevertheless, basic demographics of clinic attendees were similar to the wider RUHSA catchment population, and all villagers who met the criteria were strongly encouraged to attend for testing. The information we collected on attendees was based on self-report, with no external validation, and many study participants had low levels of education. Nevertheless, the interviewers were trained health workers with extensive experience in interacting with lower income, rural, low-health-literacy patients, using a range of strategies tailored to this population—so we have reasonable confidence in the accuracy of the data.

The surveys were conducted over discrete time periods, and there were complex background factors which are likely to have affected our results, including the timing of lockdowns and other social-distancing measures and the transmission rate of the virus (Rt) both within and outside the study region. This was shown in Tamil Nadu to vary over time and by district—likely reflecting changes in both the access to testing and compliance to preventive measures as well as the effectiveness of contact-tracing efforts [41]. Our household seroprevalence study was limited by poor response—we found a general reluctance for asymptomatic household member to provide samples, possibly relating to stigma from the illness. It did, however, suggest significant spread of the virus within the households, in keeping with international literature [39]. While we didn’t have sufficient numbers to examine within-household spread by family member characteristics, other studies have found children and adolescents to be less susceptible to COVID-19 infection but more infectious than older individuals [42]. There was disparity between some of the different tests for sero-positivity; we classified any positive test as COVID-19 sero-positive, noting that, worldwide, it’s thought we are likely to be under-, rather than over-estimating prevalence with available tests [43].

Despite its limitations, our study has provided some important insights on how the COVID-19 pandemic is playing out in a remote, rural lower-middle-income country (LMIC) population. LMIC countries are highly vulnerable to the COVID-19 pandemic, and it’s likely that additional support will be needed for communities similar to our study population which will require innovative policies to achieve sustainability and development [44]. The pandemic poses particular challenges for these communities due to the paucity of testing services, weak surveillance systems and limited access to medical care [4]. Our study population at least had the benefits of outreach health services from Christian Medical College and RUHSA—giving it advantages over other similar populations in India. The impacts of this pandemic, and especially the lockdown strategy, are multi-dimensional. Ideally, the most vulnerable populations should be systematically identified and targeted for support [45]. There are many calls for the government to assist these vulnerable communities as they meet the challenges of the pandemic [46]. COVID-19 vaccination started in India on 16th Jan 2021 but is limited to health care professionals and frontline workers at this stage—it will take some time before it reaches the rural general population. Once it does, a thorough understanding of how the pandemic is playing out in India’s lower income, rural populations will be vital in achieving efficacious and equitable national coverage [47]—for example, the vaccination threshold to achieve herd immunity may differ from populations in other regions of India.

## Conclusions

The COVID-19 pandemic is having a significant impact on lower income, rural communities in India. We’ve shown that a simple approach using community health workers, a screening instrument, and a community-based testing clinic can readily identify COVID-19 infections in a rural population. While our data suggest low rates of death and severe illness, it is vital that ongoing efforts to control the pandemic do not overlook these vulnerable populations. It is also important to examine whether available data are capturing the full impact of the disease in groups which may not be adequately represented in existing studies in India—such as the elderly, and those with multiple co-morbidities. Ongoing monitoring through seroprevalence surveys will help in predicting future patterns of the pandemic in rural communities.

## Data Availability

The datasets used and/or analysed during the current study are available from the corresponding author on reasonable request.

## Appendix 1 Screening Checklist

**Table:**

ILL: Fever (> 100.4F) and cough/other respiratory symptoms for < 10 days […]
OR
SARI: Acute respiratory illness (fever and at least one sign/symptom of Respiratory disease, e.g., cough, shortness of breath) AND history of travel to or residing in a location reporting community transmission of COVID-19 disease during the 14 days prior to symptom onset […]
OR
Patient with any acute respiratory illness having been in contact with a confirmed or probable COVID-19 case in the last 14 days prior to symptom onset […]
OR
A patient with severe acute respiratory illness (fever and at least one sign/symptom of respiratory disease, e.g., cough, shortness of breath) requiring hospitalization in the absence of an alternative diagnosis that fully explains the clinical presentation […]

## Abbreviations

RUHSA: Rural Unit of Health and Social Affairs
SES: Socio-economic status
ICMR: Indian Council of Medical Research
GoI: Government of India
CHW: Community health worker
PCR: Polymerase chain reaction
RT-PCR: Real time polymerase chain reaction
ECLIA: Electrochemiluminescence assay
CI: Confidence interval
ICR: Infection to case ratio
CMC: Christian Medical College

## References

[1] European Centre for Disease Prevention and Control. COVID-19 situation update worldwide, as of week 4, updated 4 February 2021. https://www.ecdc.europa.eu/en/geographical-distribution-2019-ncov-cases (accessed 8th February 2021).

[2] Mazumder A, Arora M, Bharadiya V, Berry P, Agarwal M, Behera P (2020). SARS-CoV-2 epidemic in India: epidemiological features and in silico analysis of the effect of. F1000Res.

[3] Mahajan P, Kaushal J (2020). Epidemic trend of COVID-19 transmission in India during lockdown-1 phase. J Commun Health.

[4] Kumar A, Nayar KR, Koya SF (2020). COVID-19: challenges and its consequences for rural health care in India. Public Health Pract.

[5] Indian Council of Medical Research. Media Report on “Briefing on COVID-19“. New Delhi, India: Department of Health Research—Ministry of Health and Family Welfare Government of India; [Last accessed on 2020 Mar 28]. https://www.icmrnicin/sites/default/files/MediaReport_COVID19pdf. [Google Scholar].

[6] Asrani P, Eapen MS, Hassan MI, Sohal SS (2021). Implications of the second wave of COVID-19 in India. Lancet Respir Med.

[7] Mahase E (2020). Covid-19: what is the R number?. BMJ.

[8] Gupta A, Banerjee S, Das S (2020). Significance of geographical factors to the COVID-19 outbreak in India. Modeling Earth Syst Environ.

[9] Ministry of Health and Family Welfare. Department of Health and Family Welfare Government of India. [Last updated on 2020 Nov 5]. Available from: https://www.mohfwgovin/.

[10] Murhekar MV, Bhatnagar T, Selvaraju S, Rade K, Saravanakumar V, Thangaraj JWV (2020). Prevalence of SARS-CoV-2 infection in India: findings from the national serosurvey, May-June 2020. Indian J Med Res.

[11] .Murhekar M, Bhatnagar T, Selvaraju S, Kumar VS, Thangaraj JW, Shah N, Santhosh Kumar M, Rade K, Sabarinathan R, Asthana S, Balachandar R. SARS-CoV-2 Antibody Prevalence in India: Findings from the Second Nationwide Household Serosurvey, 2020. Available at SSRN: https://ssrn.com/abstract=3715460 or 10.2139/ssrn.3715460.

[12] .Government of India. Letter to Chief Secretary Cabinet Secretory. New Delhi: Ministry of Health and Family Welfare Government of India; 2020. [Last accessed on 2020 Mar 28]. https://www.mohfwgovin/pdf/ChiefSecyDOLetterpdf. [Google Scholar].

[13] .World Health Organization. Critical preparedness, readiness and response actions for COVID-19: interim guidance, 22 March 2020. World Health Organization; 2020.

[14] John J, Kang G (2021). Tracking SARS-CoV-2 infection in India with serology. Lancet Glob Health.

[15] Isaac RC, Ramamurthy P, Finkel M, Kunjuvareed AI, Trevena L (2014). An educational training on cervical cancer screening program for rural healthcare providers in India. Indian J Commun Health.

[16] Government of India: Census of India, 2011: Tamil Nadu. https://censusindia.gov.in/2011census/dchb/DCHB_A/33/3304_PART_A_DCHB_VELLORE.pdf.

[17] Government of India. Ministry of Statistics and Programme Implementation. Literacy rate in India state-wise. http://mospi.nic.in/literacy-rate-india-state-wise-rgi-nsso.

[18] Sarkar S (2021). Significance of migration to the COVID 19 outbreaks in major states in India. Int J Migration Health Soc Care.

[19] Indian Council of Medical Research. Department of Health Research. Strategy for COVID19 testing in India (version 5, dated 18/05/2020) accessed online on 18th May 2020: https://www.icmr.gov.in/cteststrat.html.

[20] World Health Organisation. WHO COVID-19 : Case Definitions. Accessed online on 30th April 2020 at https://www.who.int/publications/i/item/WHO-2019-nCoV-Surveillance_Case_Definition-2020.2.

[21] Marty FM, Chen K, Verrill KA (2020). How to obtain a nasopharyngeal swab specimen. NEJM.

[22] La Marca A, Capuzzo M, Paglia T, Roli L, Trenti T, Nelson SM (2020). Testing for SARS-CoV-2 (COVID-19): a systematic review and clinical guide to molecular and serological in-vitro diagnostic assays. Reprod Biomed.

[23] World Health Organization. Population-based age stratified seroepidemiological investigation protocol for COVID-19 virus infection. Accessed online at https://apps.who.int/iris/bitstream/handle/10665/331656/WHO-2019-nCoV-Seroepidemiology-2020.1-eng.pdf?sequence=1&isAllowed=y on 27th April, 2020

[24] Government of Tamil Nadu: Open Government Data Portal of Tamil Nadu—https://tn.data.gov.in/ eGovernance Department. Electronics Corporation of Tamil Nadu (ELCOT), Chennai.

[25] Bhatt S, Isaac R, Finkel M, Evans J, Grant L, Paul B, Weller D (2018). Mobile technology and cancer screening: lessons from rural India. J Global Health.

[26] .Vellore district Health Information System. Directorate of Public Health and preventive medicine. Government of Tamil Nadu, India https://vellore.nic.in/health/.

[27] Randolph HE, Barreiro LB (2020). Herd immunity: understanding COVID-19. Immunity.

[28] .Chanda A. COVID-19 in India: transmission dynamics, epidemiological characteristics, testing, recovery and effect of weather. Epidemiol Infect. 2020; 148. 10.1017/S0950268820001776 PMC7453359 32778180

[29] Buheji M, da Costa Cunha K, Beka G, Mavric B, de Souza YL, da Costa Silva SS, Hanafi M, Yein TC (2020). The extent of covid-19 pandemic socio-economic impact on global poverty a global integrative multidisciplinary review. Am J Econ.

[30] Cao Y, Hiyoshi A, Montgomery S (2020). COVID-19 case-fatality rate and demographic and socioeconomic influencers: worldwide spatial regression analysis based on country-level data. BMJ Open.

[31] Dehingia N, Raj A (2021). Sex differences in COVID-19 case fatality: do we know enough?. Lancet Global Health.

[32] Gupta PK, Bhaskar P, Maheshwari S. Coronavirus 2019 (COVID-19) outbreak in India: a perspective so far. J Clin Exp Invest. 2020;11(4).

[33] Philip M, Ray D, Subramanian S (2021). Decoding India’s low COVID-19 case fatality rate. J Human Dev Capab.

[34] Cohen J (2021). Is India’s coronavirus death ‘paradox’ vanishing?. Science.

[35] Malani A, Shah D, Kang G, Lobo GN, Shastri J, Mohanan M, Jain R, Agrawal S, Juneja S, Imad S, Kolthur-Seetharam U (2020). Seroprevalence of SARS-CoV-2 in slums versus non-slums in Mumbai, India. Lancet Global Health.

[36] Chandramouli C, General R. Census of India 2011. Provisional Population Totals. New Delhi: Government of India. 2011: 409–13.

[37] Rostami A, Sepidarkish M, Leeflang MMG, Riahi SM, Shiadehet MN, Esfandyari S (2020). SARS-CoV-2 seroprevalence worldwide: a systematic review and meta-analysis. Clin Microbiol Infect.

[38] Madewell ZJ, Yang Y, Longini IM, Halloran ME, Dean NE (2020). Household transmission of SARS-CoV-2: a systematic review and meta-analysis. JAMA Netw Open.

[39] Haroon S, Chandan JS, Middleton J, Cheng KK (2020). Covid-19: breaking the chain of household transmission. BMJ.

[40] Ranjan R, Sharma A, Verma MK (2021). Characterization of the Second Wave of COVID-19 in India. medRxiv..

[41] Laxminarayan R, Wahl B, Dudala SR, Gopal K, Mohan C, Neelima S, Reddy KSJ (2020). Corona virus-Epidemiology and transmission dynamics of COVID-19 in two Indian states. Science.

[42] Li F, Li YY, Liu MJ, Fang LQ, Dean NE, Wong GW, Yang XB, Longini I, Halloran ME, Wang HJ, Liu PL (2021). Household transmission of SARS-CoV-2 and risk factors for susceptibility and infectivity in Wuhan: a retrospective observational study. The Lancet Infect Dis.

[43] Burgess S, Ponsford MJ, Gill D (2020). Are we underestimating seroprevalence of SARS-CoV-2?. BMJ.

[44] Burgess JC (2020). Sustainability and development after COVID-19. World Dev.

[45] Acharya R, Porwal A (2020). A vulnerability index for the management of and response to the COVID-19 epidemic in India: an ecological study. Lancet Glob Health.

[46] Harriss J (2020). Responding to an Epidemic Requires a Compassionate State: how Has the Indian State Been Doing in the Time of COVID-19?. J Asian Stud.

[47] Foy BH, Wahl B, Mehta K, Shet A, Menon GI, Britto C (2021). Comparing COVID-19 vaccine allocation strategies in India: a mathematical modelling study. Int J Infect Dis.

